# Superstructures with cyclodextrins: Chemistry and applications II

**DOI:** 10.3762/bjoc.11.30

**Published:** 2015-02-18

**Authors:** Gerhard Wenz

**Affiliations:** 1 Saarland University, Organic Macromolecular Chemistry, Campus C4 2, 66123 Saarbrücken, Germany

**Keywords:** cyclodextrins

Cyclodextrins (CDs) are cyclic α(1→4)-linked oligomeres of anhydroglucose. Since the 6-, 7- and 8-membered rings, called α-, β- and γ-CD, respectively, can be produced through the enzymatic degradation of starch on an industrial scale with high purity [1], CDs are now considered to be the most interesting class of organic host molecules. Furthermore, α- and γ-CDs are known to be nontoxic and are approved as food additives [2–3]. Since CDs are soluble in water and able to solubilize hydrophobic active ingredients, they are broadly used in biomedical applications [4]. CDs are also well known to complex suitable monomeric and polymeric guest molecules, leading to supramolecular structures and topological compounds, such as rotaxanes and polyrotaxanes [5–7]. The solubility and stability of CD complexes are controllable through the derivatization of CDs. Remarkable progress has been achieved over the past 10 years regarding the regioselective derivatization of cyclodextrins. Synthetic procedures for CD key derivatives such as mono-6-*O*-tosyl-β-CD were optimized to allow for production on a large scale [8]. More than one and up to 6 different substituents have been attached at various defined locations on the CD ring [9–10]. Some CD derivatives have already found very interesting applications in the design of aqueous-phase catalysts [11]. Many other applications are certain to follow.

The 17^th^ International Cyclodextrin Symposium, held in May 2014 in Saarbrücken, Germany provided an overview of the various aspects of CD chemistry, such as organic synthesis, analysis of inclusion compounds, polymeric and colloidal systems, as well as smart materials and drug delivery. These contributions to Cyclodextrin Chemistry are collected in this Thematic Series of the Beilstein Journal of Organic Chemistry.

Gerhard Wenz

Saarbrücken, January 2015
